# Histone Deacetylase 3 Promotes RCAN1 Stability and Nuclear Translocation

**DOI:** 10.1371/journal.pone.0105416

**Published:** 2014-08-21

**Authors:** Kyung Ah Han, Hye Seon Kang, Jee Won Lee, Lang Yoo, Eunju Im, Ahyoung Hong, Yun Ju Lee, Woo Hyun Shin, Kwang Chul Chung

**Affiliations:** Department of Systems Biology, College of Life Science and Biotechnology, Yonsei University, Seoul, Korea; Univ. Kentucky, United States of America

## Abstract

*Regulator of calcineurin 1* (*RCAN1*; also referred as *DSCR1* or *MCIP1*) is located in close proximity to a Down syndrome critical region of human chromosome 21. Although RCAN1 is an endogenous inhibitor of calcineurin signaling that controls lymphocyte activation, apoptosis, heart development, skeletal muscle differentiation, and cardiac function, it is not yet clear whether RCAN1 might be involved in other cellular activities. In this study, we explored the extra-functional roles of RCAN1 by searching for novel RCAN1-binding partners. Using a yeast two-hybrid assay, we found that RCAN1 (RCAN1-1S) interacts with histone deacetylase 3 (HDAC3) in mammalian cells. We also demonstrate that HDAC3 deacetylates RCAN1. In addition, HDAC3 increases RCAN1 protein stability by inhibiting its poly-ubiquitination. Furthermore, HDAC3 promotes RCAN1 nuclear translocation. These data suggest that HDAC3, a new binding regulator of RCAN1, affects the protein stability and intracellular localization of RCAN1.

## Introduction

Down syndrome (DS) is the most frequent genetic disorder and is caused by extra copies of all or part of chromosome 21, thus resulting in overexpression of a subset of resident genes [1]. Cytogenetic studies of patients with partial triplication have shown that the distal part of chromosome 21, called Down syndrome critical region (DSCR), is sufficient to cause many phenotypic abnormalities found in DS patients. *Regulator of calcineurin 1* (*RCAN1*; also called as *DSCR1, Adapt78, MCIP1 or calcipressin 1*) is located near DSCR and is thought to be responsible for diverse DS phenotypes [2]. DS patients display features, including mental retardation, various cardiac and gastrointestinal anomalies, immune system defects, and Alzheimer's disease [3].

RCAN1 is preferentially expressed in heart, skeletal muscle, and brain [4], and can bind to and inhibit calcineurin [5], [6]. Ca^2+^/calmodulin-dependent protein phosphatase calcineurin mediates many cellular responses including lymphocyte activation and neuronal and muscle development [7]. The RCAN1 gene consists of seven exons plus an alternative first one (exon 1 through 4) [4]. There are four possible transcripts but the major transcriptional products are isoforms that include exon 1 (RCAN1-1) or 4 (RCAN1-4). RCAN1-1 encodes a protein of 197 amino acids and is abundant primarily in fetal and adult brains [4]. A recent study revealed an additional start site upstream of exon 1, which produces RCAN1-1 with 252 amino acids [8]. In order to avoid confusion between these two products, the former is referred as RCAN1-1S (short form) and the latter as RCAN1-1L (long form).

Histone deacetylases (HDACs) catalyze the removal of acetyl groups from lysine residues in histones. They function in opposition to histone acetyltransferases (HATs) and induce transcriptional repression via chromatin condensation [9]. Studies have suggested that HDACs regulate cell cycle progression, cell proliferation, and differentiation, thereby modulating human cancer development [10], [11]. There are 18 known human histone deacetylases that are grouped into four classes based on their accessory domain structures. Class I includes HDAC1, HDAC2, HDAC3, and HDAC8. Although HDACs 1, 2, and 3 are ubiquitously expressed, HDAC3 contains an intriguingly variable C terminus, with no apparent similarity to other HDACs. In addition, HDAC3, unlike the predominantly nuclear HDACs 1 and 2, can be found in the nucleus, cytoplasm, and at the plasma membrane [12], [13]. These observations led to the hypothesis that HDAC3 may have unique properties and thus may not be completely redundant with other HDACs [9].

In addition to histones, many other nuclear proteins have been reported to be the substrates of HDACs. For example, HDAC3 can deacetylate non-histone proteins, such as MEF2 [14], NF-κB [15], and retinoblastoma protein (pRB) [16]. HDAC3 enzymatic activity can be regulated by phosphorylation/dephosphorylation [17], [18] as well as indirectly regulated through phosphorylation of its associated proteins. For instance, IκB kinase *α*-mediated phosphorylation of RelA/p65 and SMRT on NF-*κ*B-regulated promoters disrupts the complex and subsequently de-represses corresponding genes [19].

In the present study, we performed a yeast two-hybrid screen to determine RCAN1 (RCAN1-1S) binding regulators and/or its potential regulators. After screening a human fetal brain cDNA library, HDAC3 was identified as a RCAN1 binding target, suggesting that RCAN1 could be a potential substrate or functional modulator of HDAC3 or vice versa. Therefore, we examined how RCAN1 is functionally linked to HDAC3. We found that HDAC3 deacetylates RCAN1. Moreover, HDAC3 increases RCAN1 protein stability via inhibition of ubiquitin-proteasome system (UPS)-mediated RCAN1 degradation. In addition, HDAC3 induces RCAN1 nuclear translocation.

## Materials and Methods

### Materials

Dulbecco's modified Eagle's medium (DMEM), fetal bovine serum (FBS), LipofectAMINE PLUS reagent, and secondary goat anti-IgG horseradish peroxidase conjugated anti-rabbit and anti-mouse IgGs were purchased from Life Technologies (Grand Island, NY, USA). Protein A-Sepharose was obtained from GE Healthcare (Piscataway, NJ, USA). Enhanced chemiluminescence (ECL) reagent was purchased from Perkin-Elmer Life and Analytical Sciences (Waltham, MA, USA). Anti-rabbit and -mouse Flag antibodies, trichostatin A (TSA), and all other chemicals used were analytical grade commercial products purchased from Sigma-Aldrich (St. Louis, MO, USA). Anti-rabbit and -mouse HA and HDAC3 antibodies, anti-histone-H1, anti-tubulin, anti-ubiquitin, anti-Hsp90, and mouse immunoglobulins (IgGs) were purchased from Santa Cruz Biotechnology. Anti-histone and anti-acetylated lysine antibodies were purchased from Abcam and Cell Signaling, respectively. Clasto-lactacystin-lactone and MG132 were purchased from A.G. Scientific (San Diego, CA, USA). Mammalian expression vectors for HA-tagged human wild type RCAN1 (RCAN1-1S) and HA-tagged constitutively active calcineurin A (denoted as CnA or CaN) were kindly provided by S. de la Luna (Genomics Regulation Center, Barcelona, Spain) and B. A. Rothermel (University of Texas Southwestern Medical Center, Dallas, USA), respectively. The plasmid encoding Flag-tagged HDAC3 was provided by E. Seto (H. Lee Moffitt Cancer Center and Research Institute, Tampa, FL, USA). HA-tagged mammalian expression vectors encoding RCAN1 mutants with deletions spanning amino acids 1–95 (RCAN1^1-95^), 1–125 (RCAN1^1-125^), 30–197 (RCAN1^30-197^), and 90–197 (RCAN1^90–197^) were constructed as described previously [20].

### Cell culture and cell lysate preparation

Human embryonic kidney 293 (HEK293) cells were maintained in DMEM containing FBS and 100 U/ml penicillin-streptomycin. DNA transfections were performed using the Lipofectamine Plus reagent according to the manufacturer's instructions. In order to prepare cell lysates, cells were rinsed twice with ice-cold phosphate-buffered saline, and solubilized in lysis buffer (50 mM Tris, pH 7.5, containing 1.0% Nonidet P-40, 150 mM NaCl, 10% glycerol, 1 mM Na_3_VO_4_, 1 µg/ml leupeptin, 1 µg/ml aprotinin, 1 mM EGTA, 1 mM EDTA,10 mM NaF, and 0.2 mM phenylmethylsulfonyl fluoride). Cells were scraped, and supernatants were collected after centrifugation for 20 min at 13,000× *g* at 4°C.

### Immunoprecipitation and immunoblot assay

Appropriate antibodies (1 µg) were incubated overnight at 4°C with cell extracts (0.5 to 1 mg) prepared in cell lysis buffer. Protein A-sepharose beads (50 µl of 1∶1 suspension) were added and incubated for 2 hr at 4°C with gentle rotation. Beads were pelleted and washed extensively in cell lysis buffer. Immunocomplexes were dissociated by boiling in SDS-PAGE sample buffer, separated by SDS-PAGE, and transferred to nitrocellulose membranes (Millipore, Japan). Membranes were blocked for 1 hr at room temperature in TBST buffer (20 mM Tris, pH 7.5, 137 mM NaCl, and 0.1% Tween 20) plus 5% nonfat dry milk, followed by overnight incubation at 4°C in TBST buffer with 3% nonfat dry milk and the appropriate primary antibody. Membranes were washed three times in TBST, and incubated for 1 hr at room temperature with appropriate secondary IgG-coupled horseradish peroxidase antibody. Membranes were washed three times with TBST and visualized using the ECL reagent.

### Immunocytochemistry

HEK293 cells were seeded at 60% confluence onto cover glasses in P35 dishes and incubated overnight. Cells were transfected for 24 hr with HA-RCAN1 or/and Flag-HDAC3, washed with PBS, fixed for 20 min in 4% paraformaldehyde in PBS, and permeabilized for 30 min in 0.2% Triton X-100 in PBS. Cells were blocked for 30 min with 1% BSA in PBS and incubated overnight at 4°C with anti-mouse HA or anti-rabbit Flag antibodies. After washing three times with PBS, the cells were incubated for 2 h with Alexa Fluor 488-conjugated anti-mouse and Alexa Fluor 594-conjugated anti-rabbit antibodies (Molecular Probes). To stain the nuclei, cells were incubated for 5 min with 1 µg/mL DAPI in PBS. After washing with PBS three times, cells were analyzed using confocal microscopy (LSM700; Carl Zeiss).

### Preparation of Triton X-100-soluble/insoluble fractions

Cells were solubilized in 1.0% Triton X-100, and the resulting cellular suspensions were fractionated by centrifugation at 15,000× *g* for 15 min. Supernatants (*i.e.*, the Triton X-100-soluble fractions) were mixed with 5× SDS sample buffer and boiled for 5 min. The resulting pellets (*i.e.*, the Triton X-100-insoluble fractions) were sonicated for 10 sec at 25% Amp, which generates the insoluble supernatants. The samples were ice-incubated for 20 min, mixed with 5× SDS sample buffer, and boiled.

### Preparation of cytosolic and nuclear fractions

Cells were washed with ice-cold PBS and resuspended in hypotonic buffer (10 mM HEPES, pH 7.9, 1.5 mM MgCl_2_, 10 mM KCl) supplemented with protease inhibitors (including dithiothreitol, aprotinin, and leupeptin) and incubated for 30 min on ice. Cells were lysed with a disposable syringe, followed by centrifugation at 1,000× *g* for 15 min at 4°C. Supernatants were used as the cytosolic fractions. Nuclear pellet fractions were washed with hypotonic buffer, and lysed in 1.0% NP-40 lysis buffer. Supernatants from each fraction were collected after centrifugation at 15,000× *g* for 15 min at 4°C.

### Statistical analysis

Group means were compared using Student's *t*-tests. *P* values less than 0.05 were considered statistically significant.

## Results

### RCAN1 interacts with HDAC3

**Figure 1:**
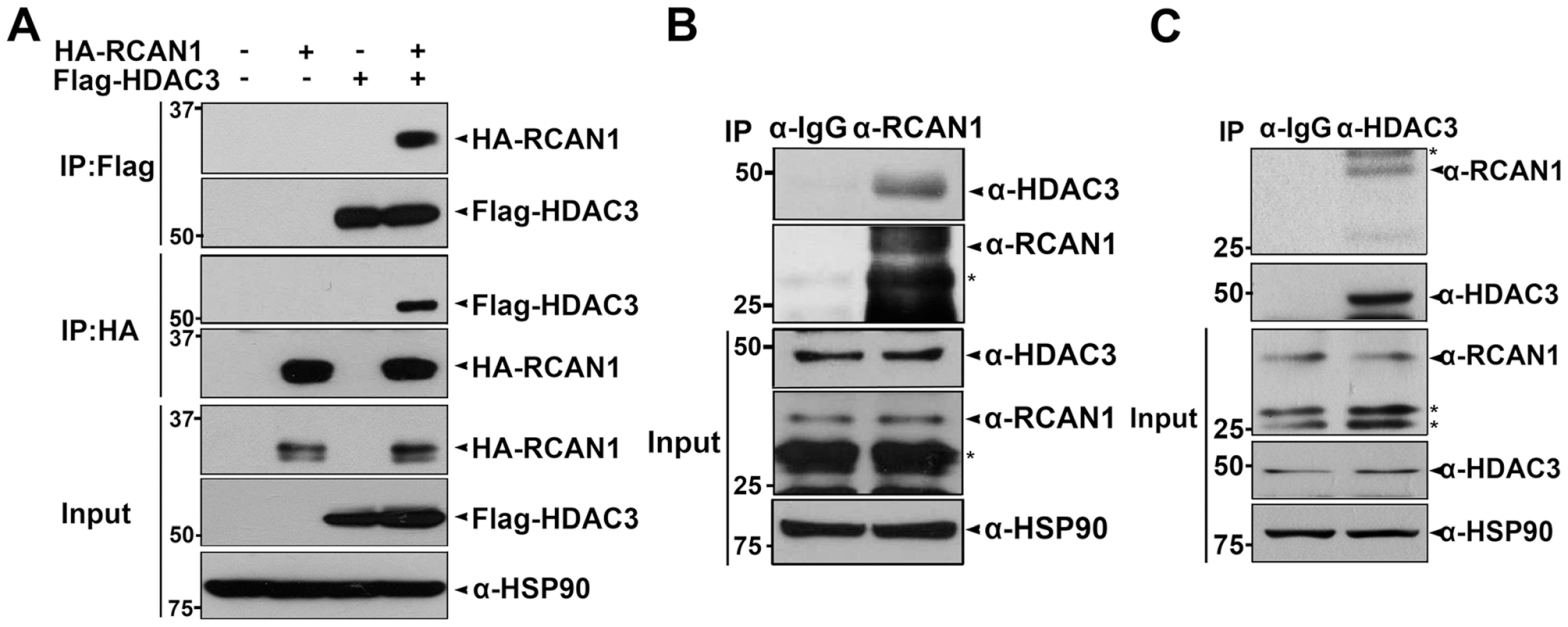
RCAN1 binds HDAC3 in HEK293 cells. (**A**) Where indicated, HEK293 cells were mock-transfected or transfected with plasmids encoding HA-tagged RCAN1 and/or Flag-tagged HDAC3 for 24 hr. Cell lysates were immunoprecipitated using anti-HA and anti-Flag antibodies, and immunocomplexes were analyzed by Western blotting using anti-HA or anti-Flag antibodies. Expression of transiently transfected proteins in cell lysates was identified using immunoblot analyses, as indicated. (**B, C**) HEK293 cell lysates were immunoprecipitated with anti-RCAN1 (**B**), anti-HDAC3 antibodies (**C**), or normal rabbit IgGs followed by immunoblotting using anti-HDAC3 and anti-RCAN1 antibodies, as indicated (*, nonspecific bands).

To investigate additional cellular roles for RCAN1, we performed a yeast two-hybrid screen of a human fetal brain cDNA library using full-length RCAN1 as bait [21]. In addition to RCAN1-binding partners including calcineurin, we identified several previously unreported binding proteins, including NF-κB-inducing kinase [21], Tollip [22], STAT2 [20], and HDAC3 (data not shown). To further evaluate the RCAN1 and HDAC3 interaction as well as to determine the functional role of this interaction, we investigated whether RCAN1 specifically associates with HDAC3 in mammalian cells. After HEK293 cells were transfected with HA-tagged RCAN1 and/or Flag-tagged HDAC3, cell extracts were immunoprecipitated with the anti-Flag or anti-HA antibody, followed by immunoblotting with the anti-HA or anti-Flag antibody. Co-immunoprecipitation assays revealed that ectopically expressed RCAN1 bound HDAC3 in HEK293 cells (Fig. 1A). Next, HEK293 cell lysates were immunoprecipitated with either preimmune IgG or anti-RCAN1 antibodies. Immunoblot analyses of the immunocomplexes using the anti-HDAC3 antibody revealed that endogenous RCAN1 associated with endogenous HDAC3 (Fig. 1B). When the co-immunoprecipitation assays were performed in a reverse order, we observed the same result (Fig. 1C). These results suggest that the RCAN1-HDAC3 interaction is not an artifact of DNA transfection but rather is a specific interaction in mammalian cells.

**Figure 2:**
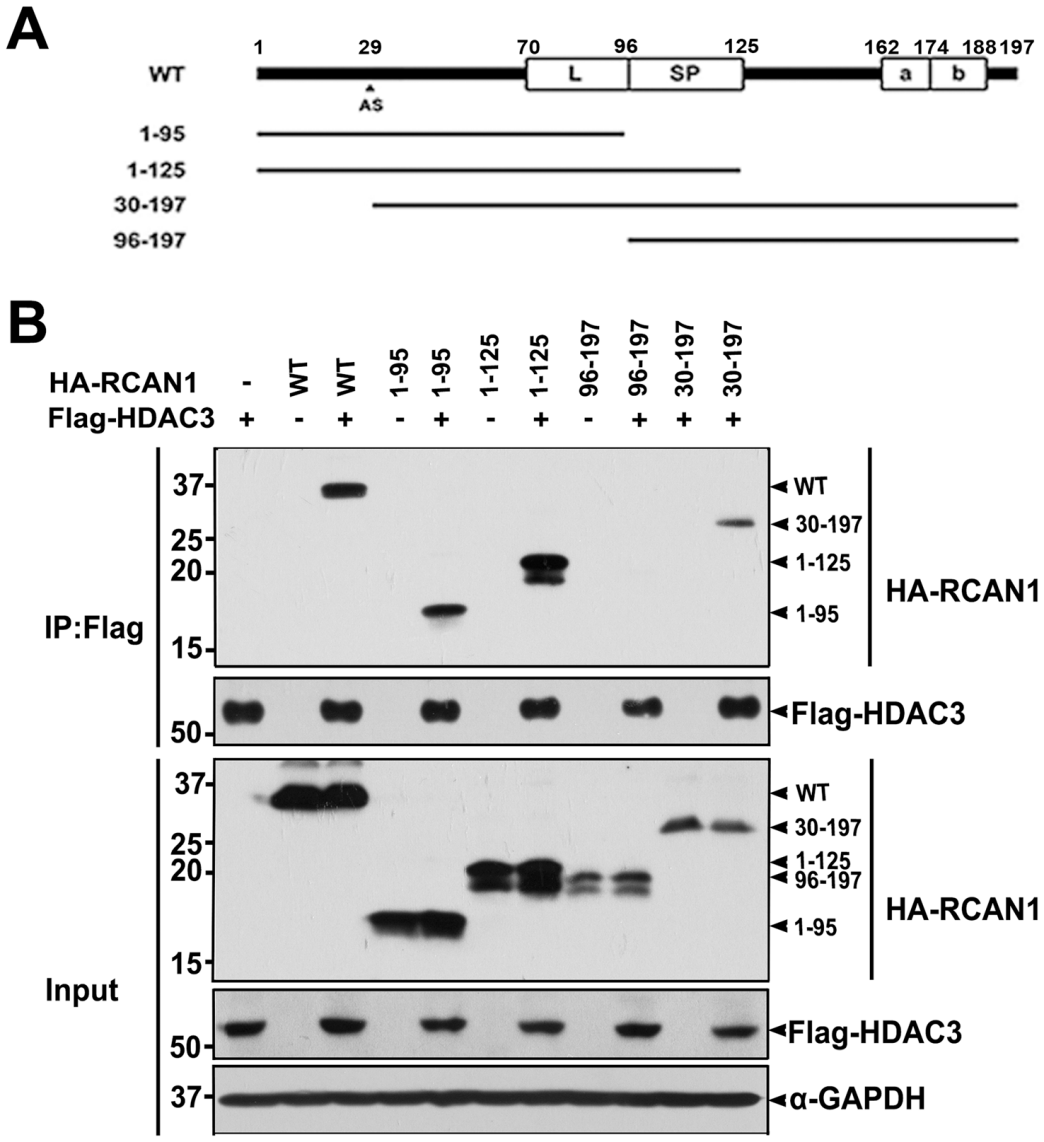
The N-terminal 30–95^th^ amino acid region of RCAN1 is critical for HDAC3 binding. (**A**) Diagram of HA-tagged wild-type RCAN1 and its deletion mutants. RCAN1 consists of an N-terminal amphipathic leucine repeat (L) domain, a central span of 31 amino acids containing a serine-proline (SP) repeat, a C-terminal acidic region (a), and a cluster of basic amino acids (b). AS denotes the alternative splicing site of *RCAN1* between exon 1 or 4 and exon 5. (**B**) HEK293 cells were transfected for 24 hr with Flag-HDAC3 alone or in combination with various HA-tagged deletion RCAN1 mutants and treated for 6 hr with 10 µM MG132, as indicated. Total lysates and anti-Flag immunoprecipitates were analyzed by immunoblot using anti-HA or anti-Flag antibodies.

To determine which domain(s) within the RCAN1 protein is responsible for the interaction with HDAC3, co-immunoprecipitation/immunoblot assays were performed using several constructs encoding RCAN1 deletion fragments fused to HA (Fig. 2A). As shown in Figure 2B, immunoblot analyses of anti-HDAC3 immunocomplexes with anti-HA IgG revealed that HDAC3 bound to full length RCAN1 as well as several RCAN1 deletion peptides, including RCAN1^1-95^, RCAN1^1-125^, and RCAN1^30–197^. However, it did not bind to RCAN1^96-197^ (Fig. 2B). This result suggests that the N-terminal amino acid region of RCAN1 from amino acid 30–95 is critical for the HDAC3 interaction.

### RCAN1 is a non-histone substrate of HDAC3

**Figure 3:**
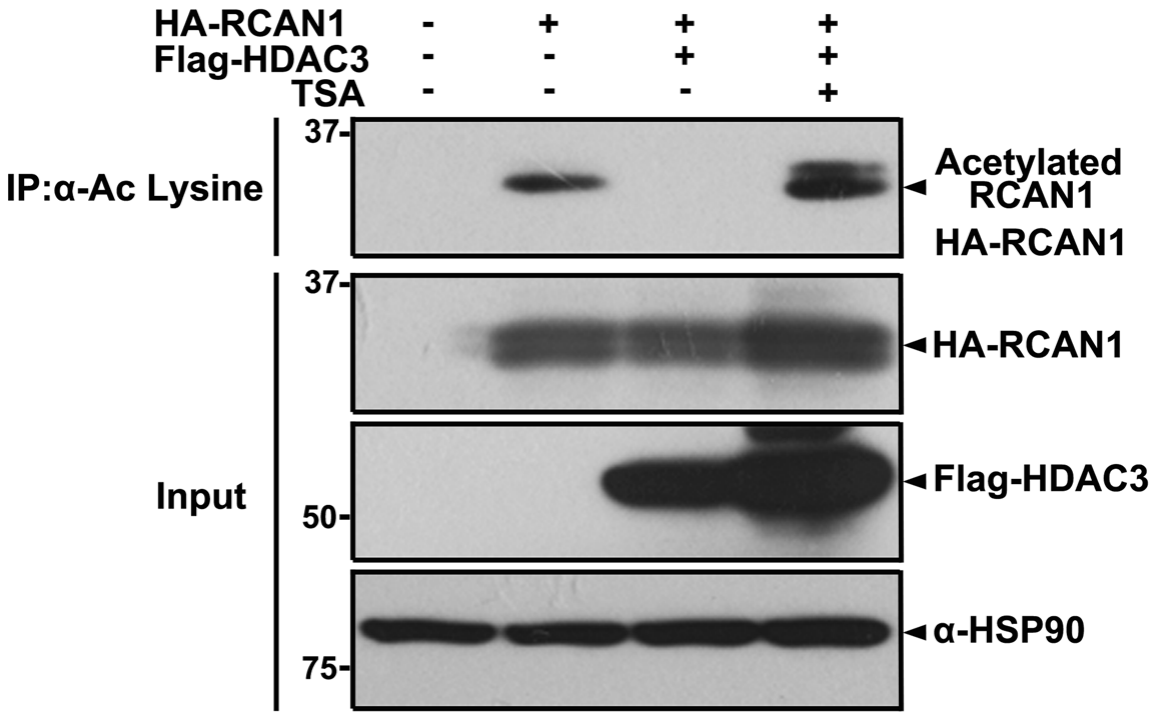
HDAC3 targets to and deacetylates RCAN1. Where indicated, HEK293 cells were transfected for 24 hr with HA-RCAN1 and/or Flag-HDAC3 (H3), and treated for 3 hr with 3 µM trichostatin A (TSA). Cell lysates were immunoprecipitated with anti-acetyl-Lys antibodies, followed by immunoblotting with anti-HA antiserum. Total lysates were analyzed by immunoblot using anti-HA or Flag antibodies. The HSP90 antibody was used as a loading control.

Next, we examined how the interaction between RCAN1 and HDAC3 affects the biochemical and functional activity of these two proteins, such as HDAC3 enzymatic activity and the inhibitory action of RCAN1 toward calcineurin A. We first checked whether RCAN1 could be a substrate of HDAC3. Co-immunoprecipitation assays of anti-acetyl-Lys immunocomplexes with the anti-HA antibody showed that exogenously expressed HA-tagged RCAN1 is considerably acetylated (Fig. 3). In addition, co-transfection of RCAN1 and HDAC3 caused a significant decrease of RCAN1 acetylation levels (Fig. 3). Moreover, compared with cells co-transfected with HDAC3 and RCAN1 alone, the presence of TSA, a HDAC class I and II inhibitor, significantly elevated RCAN1 acetylation levels (Fig. 3). Taken together, our data suggest that RCAN1 is a non-histone substrate of HDAC3.

### HDAC3 increases RCAN1 protein stability

**Figure 4:**
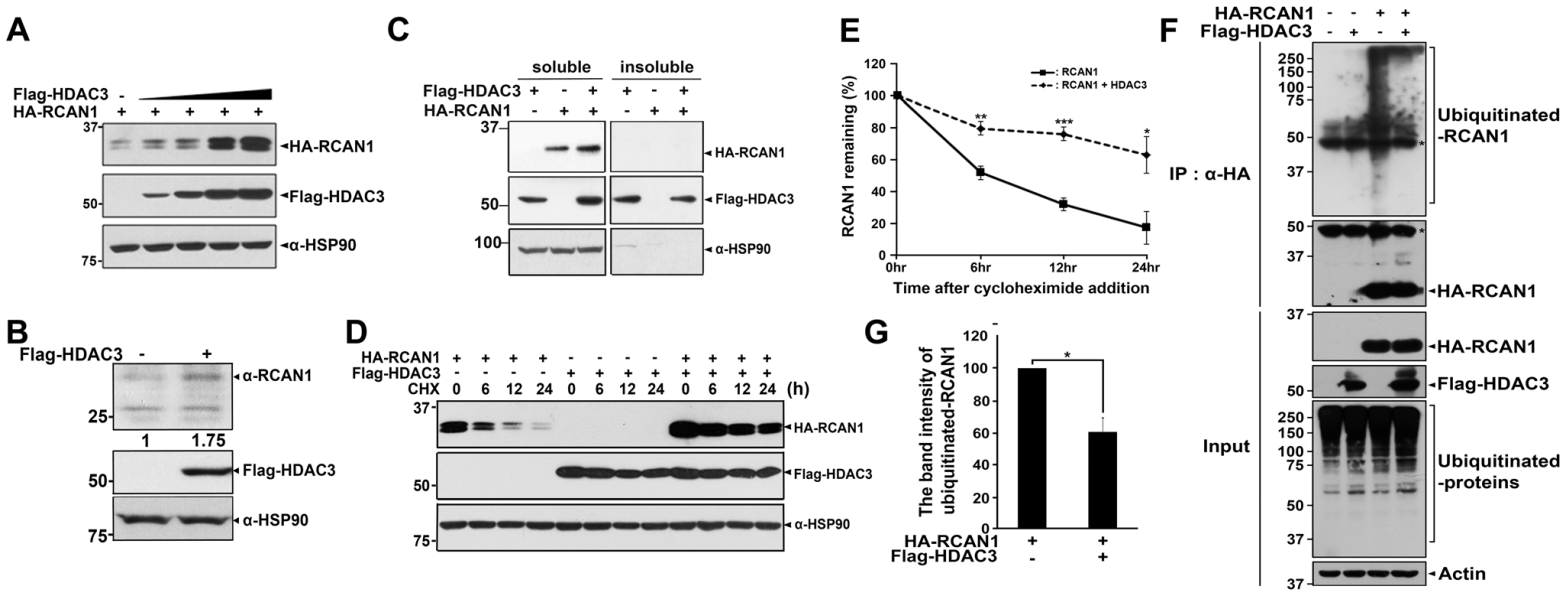
HDAC3 overexpression increases RCAN1 stability by inhibiting RCAN1 ubiquitination. (**A**) HEK293 cells were transfected for 24 hr with HA-tagged RCAN1 alone or in combination with increasing amounts of Flag-HDAC3. RCAN1 and HDAC3 levels were examined by immunoblotting with anti-HA and anti-Flag antibodies. The HSP90 antibody was used as a loading control. (**B**) HEK293 cells were mock-transfected or transfected with Flag-HDAC3 for 24 hr. Whole cell lysates were subjected to western blot analysis with anti-RCAN1, anti-Flag, and anti-HSP90 antibodies (*, nonspecific bands). (**C**) HEK293 cells were transfected for 24 hr with HA-RCAN1 and/or Flag-HDAC3 and cell extracts were lysed with Triton X-100 (soluble) and SDS (insoluble)-containing buffer. Each fraction was analyzed by immunoblotting with anti-HA or anti-Flag antibodies. To demonstrate equal loading, cell lysates were analyzed with anti-Hsp90 antibody, as indicated. (**D, E**) Where indicated, HEK293 cells were transfected for 24 hr with HA-RCAN1 or Flag-HDAC3 alone or in combination. Cells were treated with 100 µM cycloheximide (CHX) for the indicated times and harvested in PBS. RCAN1 levels in each sample were determined by western blot analyses using anti-HA antibodies (**D**). Data are representative of three independent experiments. Relative RCAN1 protein levels were quantified using the Multi Gauge V 3.1 program (*, *p*<0.05; **, *p*<0.01; ***, *p*<0.001; **E**). (**F, G**) HEK293 cells were transfected for 24 hr with HA-RCAN1 or Flag-HDAC3 alone or in combination and treated for 6 hr with 10 µM MG132, as indicated. Cell lysates were immunoprecipitated using anti-HA antibody and immunoblotted using anti-ubiquitin or anti-HA antibodies. Proper expression of ubiquitin, RCAN1, or HDAC3 in cell extracts was determined by immunoblotting with their antibodies (**F**). Data are representative of three independent experiments. Relative ubiquitinated RCAN1 protein levels were quantified using the Multi Gauge V 3.1 program (*, *p*<0.05; **G**).

Based on the finding that acetylation of Lys-ε-NH_2_ side chains and reversible deacetylation affect the biochemical and functional properties of target proteins such as their stabilities, intracellular localizations, and interactions with other proteins, we next examined whether HDAC3 affects RCAN1 protein stability or vice versa. As shown in Fig. 4A, HDAC3 co-transfection increased the steady state level of RCAN1, compared to cells transfected with RCAN1 alone. In addition, the stabilizing effect of RCAN1 was observed to be dose-dependent in HDAC3-transfected cells (Fig. 4A). Moreover, overexpressed HDAC3 caused the increase of endogenous RCAN1 levels (Fig. 4B).

Next we analyzed the effect of HDAC3 on the formation of insoluble RCAN1 aggregates. After DNA transfection, 1.0% Triton X-100-soluble and insoluble fractions were prepared from cell lysates and subjected to the immunoblotting with anti-HA antibody. As shown in the Fig. 4C, there was no insoluble RCAN1-1S aggregate after ectopic expression of RCAN1-1S alone, which is consistent with our previous finding [21]. Moreover, the presence of HDAC3 had no effect on the formation of insoluble RCAN1 aggregates, and there was no insoluble RCAN1 level (Fig. 4C). We additionally examined the effect of exogenous HDAC3 expression on the formation of endogenous RCAN1 aggregates or/and any change of its insoluble levels. These data also showed that there was no RCAN1 protein in the insoluble fraction and it remains unchanged under HDAC3 overexpression, whereas HDAC3 increases the levels of endogenous RCAN1 in soluble fraction (data not shown). These data demonstrate that the protein solubility of RCAN1 is unaffected by HDAC3.

**Figure 5:**
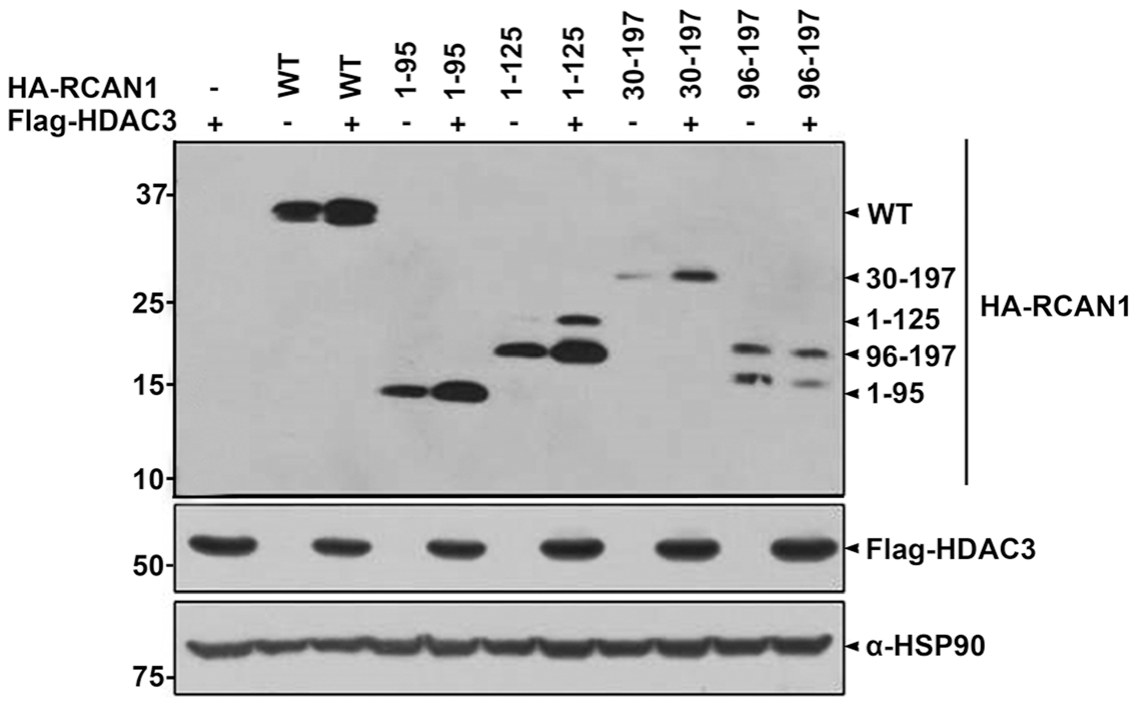
The stabilizing effect of HDAC3 is dependent on the RCAN1 N-terminal 30–95th amino acid. Where indicated, HEK293 cells were transfected for 24 hr with Flag-HDAC3 alone or together with HA-tagged wild-type RCAN1, RCAN11-95, RCAN11-125, RCAN130-197 or RCAN196-197 fragments. Cells were lysed in the lysis buffer containing 8 M urea, and immunoblot analyses were performed using HA and Flag antibodies. The HSP90 antibody was used as a loading control.

In contrast, the presence of RCAN1, as well as increasing doses of RCAN1, did not considerably affect HDAC3 levels (data not shown). Consistent with the binding pattern of RCAN1 to HDAC3, the stabilizing effect of HDAC3 toward RCAN1 was observed with wild type RCAN1, and the RCAN1^1-95^, RCAN1^1-125^, and RCAN1^30-197^ fragments, but not in the RCAN^96-197^ fragment (Fig. 5). Moreover, measurements of RCAN1 half-life using cycloheximide revealed that HDAC3 increases RCAN1 protein stability (Fig. 4D and E).

### HDAC3 inhibits the poly-ubiquitination of RCAN1

To determine the underlying mechanism regarding how HDAC3 positively regulates RCAN1 levels, we examined the effect of HDAC3 on the extent of RCAN1-ubiquitination. HEK293 cells were co-transfected with plasmids encoding either HA-RCAN1 and/or Flag-HDAC3 in the presence of MG132. Cell extracts were subsequently immunoprecipitated using the anti-HA antibody, followed by immunoblotting using the anti-ubiquitin antibody. Although ectopically expressed RCAN1 was poly-ubiquitinated, upon HDAC3 co-expression RCAN1 ubiquitination was significantly decreased (Fig. 4F and G). These results indicate that the stabilizing effect of HDAC3 on RCAN1 results from inhibition of RCAN1 ubiquitination. Therefore, in the absence of ubiquitination, RCAN1 is not targeted for proteasomal degradation.

### HDAC3 induces nuclear transport of RCAN1

**Figure 6:**
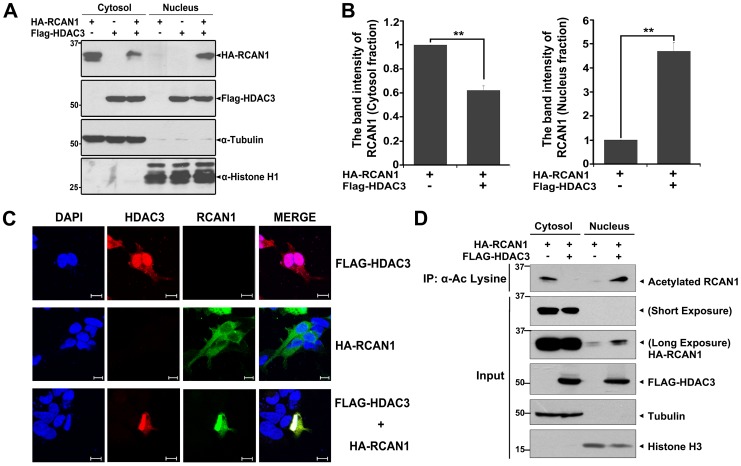
HDAC3 induces nuclear translocation of cytosolic RCAN1. (**A, B**) HEK293 cells were transfected for 24 hr with HA-RCAN1 and/or Flag-HDAC3 and fractionated into cytosolic and nuclear fractions. The fractions were analyzed by immunoblot using anti-HA or anti-Flag antibodies. The purity of each fraction was confirmed by immunoblotting with α-tubulin (cytosolic marker) or histone H1 (nuclear marker) (**A**). Data are representative of three independent experiments. Relative cytosolic RCAN1 and nuclear RCAN1 protein levels were quantified using the Multi Gauge V 3.1 program (**, *p*<0.01; **B**). (**C**) HEK293 cells were transfected for 24 hr with HA-RCAN1 or/and Flag-HDAC3, fixed and permeabilized, and labeled with anti-HA or Flag antibodies. The cells were then stained with Alexa Fluor 488-conjugated anti-mouse and Alexa Fluor 594-conjugated anti-rabbit secondary antibodies. Nuclei were counterstained with DAPI, and immunostained preparations were visualized by confocal microscopy. Scale bars: 10 µm (**D**) HEK293 cell were transfected for 24 hr with HA-RCAN1 and/or Flag-HDAC3, and fractionated into the cytosolic and nuclear fractions. These fractions were immunoprecipitated with anti-acetyl-Lys antibodies, followed by immunoblotting with anti-HA antiserum. The expression of exogenously added RCAN1 or HDAC3 protein in each fraction was analyzed by immunoblotting anti-HA or Flag antibodies. The purity of each fraction was confirmed by immunoblotting with α-tubulin (cytosolic marker) or histone H3 (nuclear marker).

We additionally evaluated whether the interaction between HDAC3 and RCAN1 affects their intracellular localization. After HEK293 cells were transfected with plasmids encoding HA-tagged RCAN1 or Flag-tagged HDAC3 alone or in combination for 24 hr, cells were fractionated into the cytoplasmic and nuclear fractions. As expected, HDAC3 was observed both in the nucleus and cytosol at a similar ratio (Fig. 6A). The presence of RCAN1 does not change the HDAC3 distribution pattern (Fig. 6A). In contrast, RCAN1 localized predominantly to the cytoplasmic compartment (Fig. 6A). Furthermore, in the presence of HDAC3, RCAN1 was observed in the nucleus, accompanied by a decrease in cytosolic RCAN1 levels (Fig. 6A and B).

Immunocytochemical analysis of HEK293 cells transfected with HA-RCAN1 also showed that overexpressed RCAN1 was mainly localized within the cytosol in the absence of HDAC3, accompanying with its small amount inside nucleus. When cells were co-transfected with RCAN1 plus HDAC3, RCAN1 was mainly present within the nucleus (Fig. 6C). These results confirmed that HDAC3 overexpression causes the nuclear translocation of RCAN1.

Next we examined whether the nuclear transported RCAN1 is deacetylated or not. HEK293 cells were transfected HA-RCAN1 or/and Flag-HDAC3 for 24 h, and fractionated into the cytoplasmic and nuclear fractions. These fractions were then immunoprecipitated with anti-acetyl-Lys antibodies, followed by the immunoblotting with anti-HA antibody. As shown in Fig. 6D, cells expressing RCAN1 alone exhibited that the acetylated RCAN1 was mainly localized in the cytosolic fraction. In addition, co-transfection of RCAN1 plus HDAC3 promoted the deacetylation of RCAN1 in the cytosolic fraction (Fig. 6D). However, co-transfection of RCAN1 and HDAC3 caused a significant increase of RCAN1 acetylation levels within the nucleus (Fig. 6D). As the increased levels of acetylated RCAN1 within the nucleus appear to be similar to the levels of nuclear-localized RCAN1, these results indicated that HDAC3-mediated nuclear translocation of RCAN1 and RCAN1 deacetylation are not linked together, but occur independently.

## Discussion

There have been reports that, in addition to histones, HDAC3 can deacetylate non-histone proteins, such as MEF2 [14], NF-κB [15], and pRB [16]. HDAC3 also interacts with many nuclear and cytosolic proteins, including nuclear receptor co-repressors 1 and 2, the zinc finger transcription factor YY1, GATA1 and 2, RELA, peroxisome proliferator-activated receptor-γ and -δ, MAPK11, cyclin D1, RUNX2, and ubiquitin [24]. Here we report that HDAC3 physically and functionally interacts with RCAN1. Interestingly, exogenously expressed RCAN1 proteins were highly acetylated in resting condition, and it becomes a non-histone substrate of HDAC3. Since lysine side chains are cationic at physiological pH, N-acetylation of Lys-ε-NH_2_ side chains would quench the positive charges, whereas deacetylation produces the positive charge again. Alternatively, the acetyl groups would provide additional link and acetylated lysine side chains can be specifically recognized, for example by bromodomains in partner proteins. Like other HAT/HDAC substrates, deacetylation also changes RCAN1 protein stability and intracellular address.

RCAN1 biochemical and functional activity is regulated by many types of post-translational modification modes. The most prominent regulatory mechanism is phosphorylation, and a number of protein kinases negatively or positively regulate RCAN1. For example, phosphorylation of the FLISPP motif within RCAN1 increases the calcineurin-inhibition effect of RCAN1 [8]. In addition, PKA and Dyrk1A increase the calcineurin inhibitory effect of RCAN1 [25], [26]. In contrast, the MEK5-MEKK3-BMK signaling cascade, GSK-3β, and TAK1 phosphorylate RCAN1 and suppress its inhibition effect on calcineurin activity [23], [27]–[29]. In addition to phosphorylation, RCAN1 was shown to be a target of ubiquitin and ubiquitin-like modifiers. Recently, we have shown that covalent conjugation of NEDD8 to RCAN1 increases the inhibitory effect of RCAN1 to the calcineurin-NF-AT pathway [30]. Furthermore, several reports have attempted to decrease RCAN1 expression levels via activation of its degradation signaling. Stimulation of H_2_O_2_-induced SCFβ-TrCP-mediated ubiquitination of RCAN1 resulted in RCAN1 degradation [31]. In addition, CREB, depending on its transcriptional activation, was also reported to activate RCAN1 degradation [32]. Our results show that RCAN1 is a target of HDAC3-mediated deacetylation, which consequently increases RCAN1 protein stability. In a similar way, class I HDACs regulate substrate protein stabilities. For example, HDAC3-mediated deacetylation increases cyclin A stability [33]. HDAC1 also deacetylates HIF-1 at Lys-709 residue, which results in the decrease of HIF-1 stability [34].

RCAN1 protein levels are also regulated by several degradation pathways, including the UPS and lysosomal pathway [32], [35], [36]. Specifically, FBW7 [37], SCFβ-TrCP [31], and Nedd4-2 [38] have been reported to covalently modify RCAN1 through poly-ubiquitination to trigger RCAN1 degradation. We have previously shown that STAT2 facilitates ubiquitin-dependent RCAN1 degradation and its action is mediated through FBW7 E3 ligase [22]. Furthermore, we demonstrated that FBW7-mediated RCAN1 degradation is enhanced by IFN-α treatment [22]. Based on these reports, it would be interesting to examine whether HDAC3 affects these enzymes. We also recently demonstrated that similar to STAT2, CREB can activate RCAN1 degradation, depending on its transcriptional activation [32]. Like the UPS, the chaperone-mediated autophagy pathway can also degrade RCAN1 [36].

The present study also revealed that HDAC3 promotes RCAN1 nuclear translocation. According to a previous report, the C-terminal 33 amino acid region of RCAN1 is important for nuclear localization [39]. It has been also reported that phosphorylation of the Thr-192 residue changes RCAN1 localization [26]. Regarding HDAC3, its deacetylase activity is critical for its modulatory action of intracellular substrate localization. For example, HDAC3-mediated deacetylation of RelA, a subunit of NF-κB promotes its nuclear export, which is depends on IκB during ES cell-derived endothelial cell differentiation [40]. In addition, acetylation of eIF5A [41], POP-1 [42], pRB [43], and PKM2 [44] changes the intracellular localization of protein. These examples suggest that HDAC3-mediated deacetylation may also trigger RCAN1 nuclear transport. However our results showed that the nuclear-localized HDAC3 is not deacetylated, suggesting that HDAC3-mediated nuclear transport of RCAN1 occurs irrelevant of its deacetylase activity. The biological and physiological roles of RCAN1 within the nucleus have not been clarified yet. Moreover, the detailed mechanism regarding when and how cytosolic RCAN1 is translocated into nucleus and vice versa is largely unknown, and more experiments are required to answer these questions.

In summary, the present work shows that HDAC3 and RCAN1 interact. RCAN1 is a deacetylation substrate of HDAC3. In addition, HDAC3 inhibits RCAN1 degradation via UPS as well as stimulates its nuclear translocation.
